# The maximum standardized uptake value in patients with recurrent or persistent prostate cancer after radical prostatectomy and PSMA-PET-guided salvage radiotherapy—a multicenter retrospective analysis

**DOI:** 10.1007/s00259-022-05931-5

**Published:** 2022-08-19

**Authors:** Simon K. B. Spohn, Andrea Farolfi, Sarah Schandeler, Marco M. E. Vogel, Juri Ruf, Michael Mix, Simon Kirste, Francesco Ceci, Stefano Fanti, Helena Lanzafame, Francesca Serani, Christian Gratzke, August Sigle, Stephanie E. Combs, Denise Bernhardt, Juergen E. Gschwend, Josef A. Buchner, Christian Trapp, Claus Belka, Peter Bartenstein, Lena Unterrainer, Marcus Unterrainer, Matthias Eiber, Stephan G. Nekolla, Kilian Schiller, Anca L. Grosu, Nina-Sophie Schmidt-Hegemann, Constantinos Zamboglou, Jan C. Peeken

**Affiliations:** 1 Department of Radiation Oncology, University Medical Center Freiburg, Faculty of Medicine, University of Freiburg, Robert-Koch-Straße 3, 79106 Freiburg, Germany; 2 German Cancer Consortium (DKTK), Partner Site Freiburg, Freiburg, Germany; 3 Berta-Ottenstein-Programme, Faculty of Medicine, University of Freiburg, Freiburg, Germany; 4 Nuclear Medicine, IRCCS Azienda Ospedaliero-Universitaria Di Bologna, Bologna, Italy; 5 Department of Radiation Oncology, Klinikum Rechts Der Isar, Technical University of Munich, Munich, Germany; 6 German Cancer Consortium (DKTK), Partner Site Munich, Munich, Germany; 7 Department of Nuclear Medicine, Faculty of Medicine, Medical Center, University of Freiburg, Freiburg, Germany; 8 Division of Nuclear Medicine, IEO European Institute of Oncology Scientific IRCCS, Milan, Italy; 9 Department of Oncology and Hemato-Oncology, University of Milan, Milan, Italy; 10 Department of Urology, University Medical Center Freiburg, Faculty of Medicine, University of Freiburg, Freiburg, Germany; 11 Institute of Radiation Medicine, Helmholtz Zentrum München, Munich, Germany; 12 Department of Urology, Klinikum Rechts Der Isar, Technical University of Munich, Munich, Germany; 13 Department of Radiation Oncology, University Hospital, LMU Munich, Munich, Germany; 14 Department of Nuclear Medicine, University Hospital, LMU Munich, Munich, Germany; 15 Department of Nuclear Medicine, Klinikum Rechts Der Isar, Technical University of Munich, Munich, Germany; 16 German Oncology Center, European University of Cyprus, Limassol, Cyprus

**Keywords:** PSMA-PET, Salvage radiotherapy, SUVmax, Risk stratification, Personalization

## Abstract

**Purpose:**

This study aims to evaluate the association of the maximum standardized uptake value (SUVmax) in positron-emission tomography targeting prostate-specific membrane antigen (PSMA-PET) prior to salvage radiotherapy (sRT) on biochemical recurrence free survival (BRFS) in a large multicenter cohort.

**Methods:**

Patients who underwent ^68^ Ga-PSMA11-PET prior to sRT were enrolled in four high-volume centers in this retrospective multicenter study. Only patients with PET-positive local recurrence (LR) and/or nodal recurrence (NR) within the pelvis were included. Patients were treated with intensity-modulated-sRT to the prostatic fossa and elective lymphatics in case of nodal disease. Dose escalation was delivered to PET-positive LR and NR. Androgen deprivation therapy was administered at the discretion of the treating physician. LR and NR were manually delineated and SUVmax was extracted for LR and NR. Cox-regression was performed to analyze the impact of clinical parameters and the SUVmax-derived values on BRFS.

**Results:**

Two hundred thirty-five patients with a median follow-up (FU) of 24 months were included in the final cohort. Two-year and 4-year BRFS for all patients were 68% and 56%. The presence of LR was associated with favorable BRFS (*p* = 0.016). Presence of NR was associated with unfavorable BRFS (*p* = 0.007). While there was a trend for SUVmax values ≥ median (*p* = 0.071), SUVmax values ≥ 75% quartile in LR were significantly associated with unfavorable BRFS (*p* = 0.022, HR: 2.1, 95%CI 1.1–4.6). SUVmax value in NR was not significantly associated with BRFS. SUVmax in LR stayed significant in multivariate analysis (*p* = 0.030). Sensitivity analysis with patients for who had a FU of > 12 months (*n* = 197) confirmed these results.

**Conclusion:**

The non-invasive biomarker SUVmax can prognosticate outcome in patients undergoing sRT and recurrence confined to the prostatic fossa in PSMA-PET. Its addition might contribute to improve risk stratification of patients with recurrent PCa and to guide personalized treatment decisions in terms of treatment intensification or de-intensification.

This article is part of the Topical Collection on Oncology—Genitourinary.

**Supplementary Information:**

The online version contains supplementary material available at 10.1007/s00259-022-05931-5.

## Introduction

Up to 50% of patients with localized prostate cancer (PCa) undergoing radical prostatectomy (RPE) experience biochemical relapse within the first 5 years after treatment [1–3]. Early salvage radiation therapy (sRT) with or without androgen deprivation therapy (ADT) is recommended as the only curative treatment option [4]. However, recurrence rate after sRT is represented by heterogeneous patterns and influenced by clinico-pathological features such as pre-treatment prostate-specific antigen values (PSA), International Society of Urological Pathology Grade (ISUP), extracapsular extension (ECE), and seminal vesicle infiltration (SVI) and surgical margins with progression-free survival rates vary between 20 and 70% after 5 years [2, 5]. Therefore, additional prognostic markers are needed to improve risk stratification and guide personalized treatment approaches such as dose escalation, adaption of RT fields, or intensification of systemic treatments. Implementation of positron emission tomography (PET) targeting the prostate-specific membrane antigen (PSMA), a cell-surface transmembrane protein over-expressed in PCa cells [6], improves detection rates of recurrent PCa lesions even at low PSA levels and outside the prostatic fossa [7, 8]. These findings have high impact on the management of salvage treatments with alteration in approximately half of patients [9–11]. While retrospective evidence supports putative improvements of biochemical recurrence free survival rates due to PSMA-PET-guided sRT [12, 13], results of prospective trials are pending ([14], NCT04794777, PATRON–NCT04557501). Emerging data suggest that the maximum standardized uptake value (SUVmax) can be used as a biomarker to prognosticate clinically significant PCa [15], Gleason-Score (GS) [16, 17], and distant metastases [18] in primary PCa, but no data exist on the applicability of this PSMA-PET feature in patients with relapse undergoing sRT. In search of new, non-invasive biomarkers for personalized risk stratification, this retrospective multicenter study aims to evaluate the impact of the SUVmax on biochemical recurrence free survival (BRFS) in patients with recurrent or persistent PCa cancer after RPE and ^68^ Ga-PSMA11-guided salvage radiotherapy.

## Methods

### Patients and treatment

**Fig. 1:**
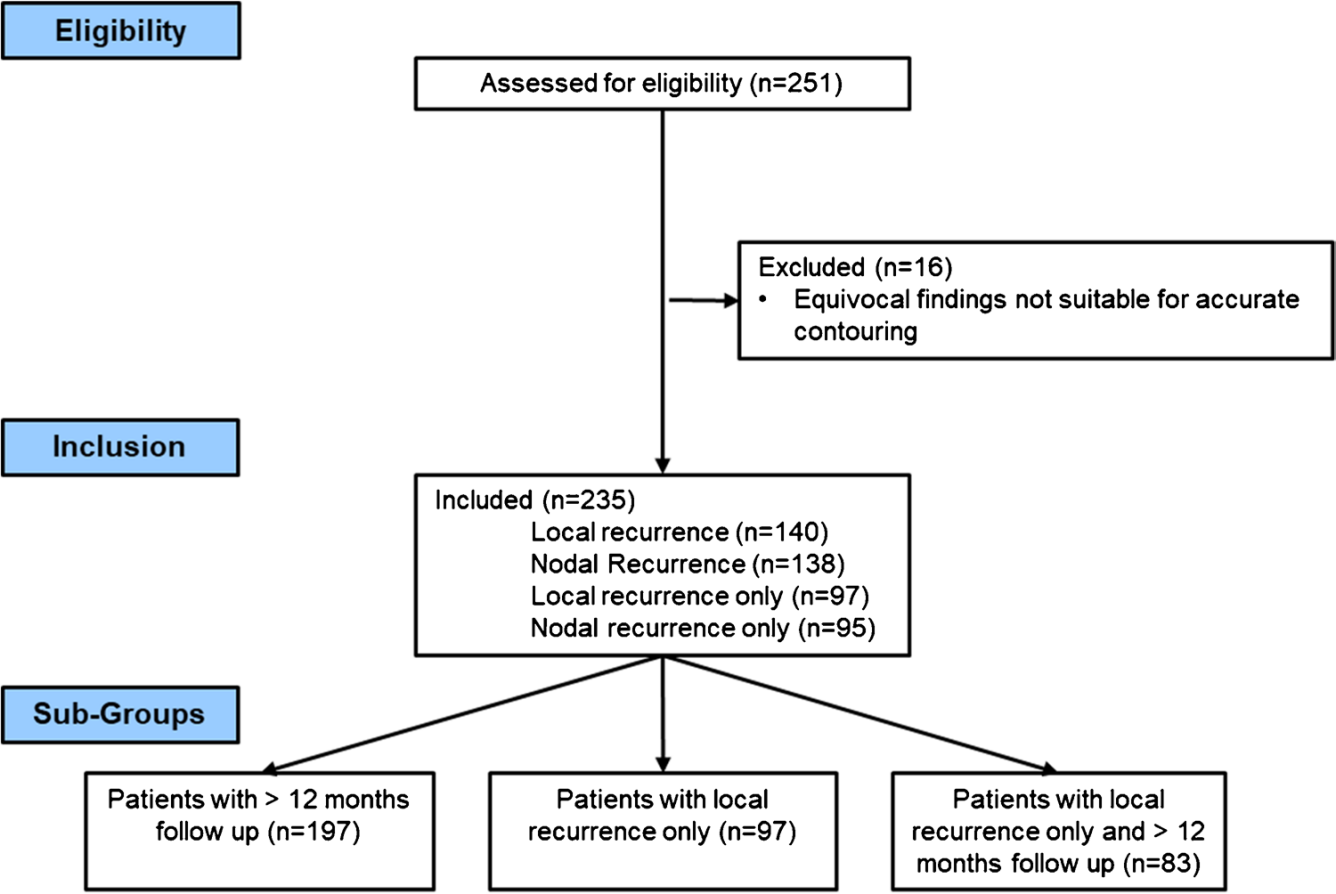
Consort flow diagram

This multicenter study collected from high-volume centers in Germany (University Medical Centre Freiburg, Klinikum Rechts der Isar Technical University Munich (TUM), University Hospital of the Ludwig-Maximillian’s-University Munich (LMU)) and Italy (IRCCS Azienda Ospedaliero-Universitaria di Bologna). The study received institutional review board approval from all participating institutions (Freiburg No.: 15/18; TUM:466/16 S; Bologna: 385/2021/Oss/AOUBo, LMU: 17–765). The centers collected data from patients who received radical surgery and underwent ^68^ Ga-PSMA11-PET due to PSA persistence (PSA after surgery ≥ 0.1 ng/ml) or recurrence (PSA ≥ 0.2 as nadir after surgery) and were subsequently treated with PSMA-PET-guided sRT. Treatment decisions were taken locally at the discretion of the treating physicians according to standards of care at the time of treatment [4] and based on PSMA-PET/CT findings. RT to the prostatic fossa was not omitted in case of nodal recurrence (NR) only. See supplementary Table 1 for details on salvage RT concepts. ADT was administered at the discretion of the treating physician. Patients were excluded if distant metastases (lymph nodes above the iliac bifurcation, bone metastases, or visceral metastases) were present in PSMA PET/CT and if ADT was given prior to PSMA PET/CT scans. Two hundred and fifty-one patients with local recurrence (LR) and/or nodal recurrence (NR) treated with sRT between 2014 and 2020 met initial inclusion criteria. Sixteen patients were excluded due to equivocal PET findings not suitable for accurate contouring of PET-lesions, resulting in two hundred and thirty-five patients in the final cohort. Additionally, a subgroup of patients with follow-up time > 12 months was created (*n* = 197). See Fig. 1 for a consort flow diagram.

### ^68^Ga-PSMA11 PET and images analysis

^68^ Ga-PSMA11 was synthesized according to good manufacture practice in all centers and in accordance with international procedural guidelines [19]. PET/CT images were acquired approximately 60 min after tracer injection (approximately 1.8–2.2 MBq ^68^ Ga-PSMA11 per kg bodyweight) in all centers and for the PSMA PET/CT contrast-enhanced or unenhanced CTs using a slice thickness of 2 mm 120 kVp, 100–400 mAs, and dose modulations were performed for attenuation correction. The following scanners were used: Freiburg: 16-slice Gemini TF Big Bore, 64-slice Gemini TF or Vereos, all Philips Healthcare, USA; TUM: Biograph mCT/128 slice CT, Siemens Healthineers, Germany; LMU: Biograph 64 and Biograph mCT (Siemens Healthineers, Germany) or Discovery 690 (GE Healthcare, USA); Bologna: Discovery MI or Discovery 710 (both GE Healthcare, USA). All scanners fulfilled the requirements indicated in the European Association of Nuclear Medicine (EANM) imaging guidelines and obtained EANM Research Ltd. (EARL1) accreditation during acquisition.

The following reconstruction algorithms were used: Freiburg: Gemini TF 64 and Gemini TF BigBore: LOR-based ordered-subset iterative time-of-flight algorithm using spherical coordinates (BLOB-OS-TF) with 3 iterations and 33 subsets and a relaxation parameter for smoothing. Vereos: BLOB-OS-TF with 3 iterations and 9 subsets without smoothing [20]; TUM: The reconstruction algorithm included point-spread-function and time-of-flight with 3 iterations and 21 subsets; LMU: Biograph 64: TrueX (3 iterations, 21 subsets) with Gaussian post-reconstruction smoothing (2 mm full width at half-maximum). Biograph mCT: TrueX (3 iterations, 21 subsets). Discovery 690: VUE Point Fx algorithm with 2 iterations and 36 subsets; Bologna: Discovery MI: time-of-flight, 8 subsets and 4 iterations; Discovery 710: time-of-flight, 18 subsets and 3 iterations.

All systems resulted in a PET image with a voxel size of 2 × 2 × 2 mm^3^. Images were normalized to decay corrected injected activity per kg body weight (SUV g/ml).

All PSMA-PET images were locally reviewed prior to data sharing by two nuclear medicine physicians with experience on PCa imaging and according to reporting international guidelines [21, 22]. Disagreements were resolved by consensus.

### Image processing

Image analysis was performed with 3D Slicer v4.10.0 [23]. Considering the local nuclear medicine report, PSMA-PET-positive LR and NR lesions were manually contoured by one reader (SS) with > 3 years’ experience in PSMA-PET segmentation using a window level from SUVmin-max: 0–5 based on previous windowing recommendations in primary PCa patients [24]. Under consideration of CT images and available PSMA-PET/CT results, any focal uptake higher than adjacent background in more than one slice was considered to represent PCa. Equivocal or small findings limited to one slice were not segmented. SUVmax was extracted for each lesion separately. Since segmentation of LR adjacent to the bladder wall can be challenging, an inter-observer variability analysis was performed by a second experienced reader (CZ) in a subset of 15 cases.

### Data collection and follow-up

Data collection included age at sRT, International Society of Urologic Pathology Grading (ISUP), pathological T-, N-stage and status of surgical margins after RPE, PSA prior to sRT, site of recurrence (local, nodal or both), and administration and duration of ADT and sRT doses. Follow-up assessments included serum PSA testing at regular intervals based on the institutional clinical praxis.

### Statistical analysis

The primary study endpoint was BRFS, defined as serum PSA > 0.2 ng/ml above the post-sRT nadir without initiation of additional salvage therapies or death of any cause. Descriptive statistics were performed with Excel 2016 (Microsoft Cooperation, USA) and GraphPad Prism v8.4.2 (GraphPad Software Inc, USA). Uni- and multivariate Cox-regression was performed with SPSS v27.0 (IBM, USA) to assess the impact of the different variables on BRFS.

Variables were dichotomized: ISUP < 3 and ≥ 3, pathological T stage < pT3 and ≥ pT3, pathological N stage pN + and pN-, positive surgical margin vs negative surgical margin, pre-sRT PSA < 0.5 ng/ml and > 0.5 ng/ml, presence and absence of local recurrence, presence and absence of nodal recurrence, administration or omission of ADT, RT to the pelvics, and dose delivered to the prostatic fossa/local recurrence (< and ≥ 72 Gy (*α*/*β* = 1.6 Gy)). Due to missing established threshold values, SUVmax values dichotomized < median and ≥ median as well as < 75% quartile (third quartile) and ≥ 75% quartile of the values of the cohort. Kaplan–Meier survival curves compared by log-rank test (GraphPad Prism v8.4.2, GraphPad Software Inc, USA) were used for analysis of the respective parameters. Thresholds of median and 75% quartile SUVmax values were applied separately for LR and NR lesions. The respective threshold of the whole cohort was applied for subgroup analysis. Time-dependent receiver-operating-characteristics (ROC) analysis was performed using R v4.1.2 [25]. Maximally selected rank statistic optimized for the log-rank test using R v4.1.2 [25] was performed to determine an optimal cut-off value for SUVmax.

## Results

### Patient characteristics

Two hundred thirty-five patients (Freiburg *n* = 39, TUM *n* = 56, LMU *n* = 64, Bologna *n* = 76) were included in the final analysis. Ninety-seven patients had LR only, 95 patients LN only, and 43 patients LR and NR in PSMA-PET. Fifty-one percent of patients received ADT. Fifty-nine percent of these patients received ADT over a duration of ≤ 12 months. Median follow-up was 24 months (IQR 16–41 months). No patient died during FU. See Table 1 for details.

**Table 1:** Patient characteristics

Cohort	All		> 12-month follow-up	
Number	235		197	
**Age (range)**	71 (65–75)		71 (65–75)	
**ISUP grade**				
1 + 2	45	19%	34	17%
3–5	185	79%	159	81%
n/a	5	2%	4	2%
**pT-stage**				
2a–c	75	32%	61	31%
3–4	134	57%	112	57%
n/a	26	11%	24	12%
**Resection stage**				
R0	98	42%	80	41%
R1	73	31%	66	34%
n/a	64	27%	51	26%
**pN-stage**				
pN0	130	55%	108	55%
pN1	51	22%	42	21%
n/a	54	23%	47	24%
**PSA prior sRT**				
< 0.5 ng/ml	66	28%	57	29%
≥ 0.5 ng/ml	164	70%	137	70%
n/a	5	2%	3	2%
**LR in PSMA-PET**	140	60%	117	59%
**NR in PSMA-PET**	138	59%	114	58%
**LR only in PSMA-PET**	97	41%	83	42%
**NR only in PSMA-PET**	95	40%	80	41%
**LR and NR in PSMA-PET**	43	18%	34	17%
**RT to the prostatic fossa/local recurrence**(*α/β = 1.6 Gy)*				
< 70 Gy	162	69%	139	71%
≥ 70 Gy	33	14%	24	12
≥ 72 Gy	38	16%	32	16%
n/a	2	1%	2	1%
**RT to elective pelvics**	154	60%	125	63%
n/a	31	13%	24	12%
**ADT**	120	51%	99	50%
Of which > 12 months	49	41%	44	44%
Of which ≤ 12 months FU	71	59%	55	56%

Abbreviation: *ISUP*, International Society of Urological Pathology; *pT*, pathological T stage; *pN*, pathological nodal stage; *n/a*, not available; *PSA*, prostate-specific antigen; *sRT*, salvage radiotherapy; *LR*, local recurrence; *NR*, nodal recurrence; *PSMA-PET*, positron-emission tomography targeting prostate-specific membrane antigen; *ADT*, androgen deprivation therapy

Median SUVmax for LR and NR was 7.6 (IQR 5.3–12.8) and 7.8 (IQR 4.3–17.5), respectively. The inter-observer analysis of 15 patients revealed significantly different volumes of manually segmented PET-positive LR lesions (median 2.7 ml (IQR 1.8–7.3 ml) vs 1.2 ml (IQR 0.5–4.1 ml), *p* < 0.001) but no significant differences between SUVmax values. See supplementary Fig. 1 for details.

### Cox-regression

**Fig. 2:**
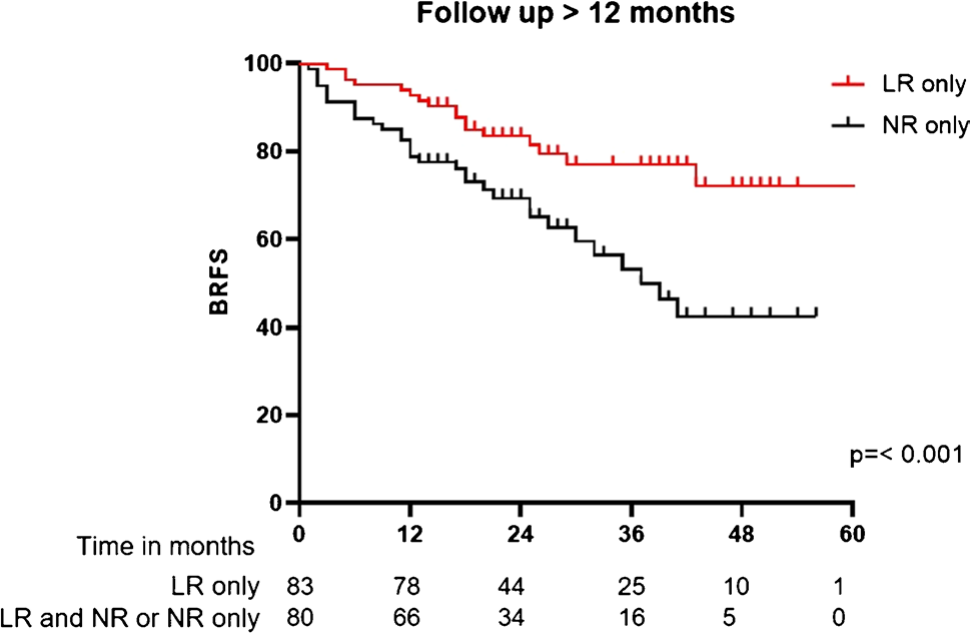
Kaplan–Meier curves for biochemical recurrence free survival (BRFS) for patients with local recurrence (LR) only and nodal recurrence (NR) only. Statistical comparison was performed with log-rank test

**Fig. 3:**
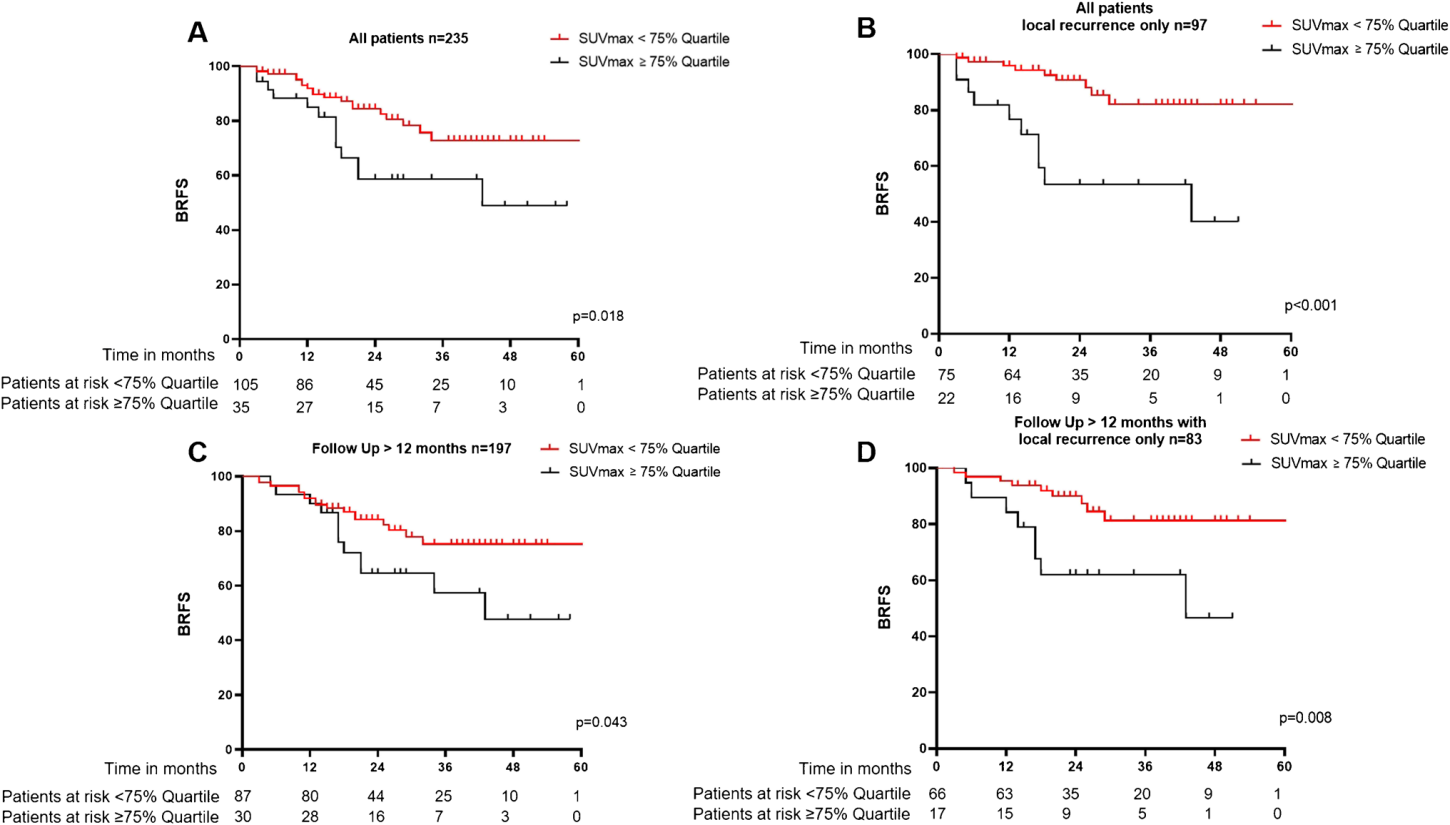
Kaplan–Meier curves for BRFS according to SUVmax values in different subgroups. Kaplan–Meier curves are represented for the impact of maximal standardized uptake values (SUVmax) ≥ 75% quartile or < 75% quartile in local recurrences of the entire cohorts’ values (A) on biochemical recurrence free survival (BRFS). Additionally results for all patients with local recurrence only (B), patients with a follow-up of > 12 months (C) and follow-up of > 12 months and local recurrence only (D) are shown.Statistical comparison was performed with log-rank test

Two-year BRFS for all patients was 68%. For patients with LR and NR only, 2-year BRFS was 80% and 65%, respectively. See Fig. 2 for Kaplan–Meier-curves. In univariate analysis, established clinical and histopathological parameters of RPE were not significantly associated with BRFS. In univariate analysis, presence of LR (*p* = 0.016, HR 0.5 95%CI 0.3–0.9) and ADT (*p* =  < 0.001, HR 0.4 95%CI 0.2–0.7) was associated with more favorable BRFS and the presence of NR with unfavorable BRFS (*p* = 0.007, HR 2.1 (95%CI 1.2–3.5). In LR lesions, there was a trend for association of values ≥ median SUVmax (7.8) and unfavorable BRFS (*p* = 0.071), while SUVmax values ≥ 75% quartile (12.8) were significantly associated with unfavorable BRFS (*p* = 0.022, HR 2.3) (95%CI 1.1–4.6). In NR lesions, no significant association of values ≥ median or 75% of SUVmax (17.5) was observed. To further assess whether SUVmax values in LR are associated with BRFS, we performed a subgroup analysis with patients who only had LR (*n* = 97). This analysis showed again no significant association of classical clinical and histopathological parameters with BRFS, but a trend for association of values ≥ median SUVmax with unfavorable BRFS (*p* = 0.05, HR 2.6, 95%CI 1.0–6.9), while SUVmax values ≥ 75% quartile were significantly associated with unfavorable BRFS (*p* = 0.001, HR 4.6, 95%CI 1.9–11.5). ADT was not associated with BRFS in this cohort. See Table 2 and Fig. 3 for details.

**Table 2:** Univariate and multivariate Cox-regression

Endpoint: BRFS									
Cohort	All (*n* = 235)			Only local recurrence (*n* = 97)		All > 12 months follow-up (*n* = 197)		Only local recurrence > 12 months follow-up (*n* = 83)	
	Variable	*n* = 235 *p*-value	HR (95%CI)	*p*-value	HR (95%CI)	*p*-value	HR (95%CI)	*p*-value	HR (95%CI)
Univariate	ISUP	0.139	1.7 (0.8–3.5)	0.198	2.3 (0.7–7.7)	0.089	2.0 (0.9–4.4)	0.311	1.9 (0.5–6.7)
	pT-stage	0.104	1.6 (0.9–2.7)	0.179	2.0 (0.7–5.6)	0.053	1.8 (1.0–3.3)	0.150	2.3 (0.7–7.0)
	pN-stage	0.476	1.2 (0.7–2.1)	0.799	0.8 (0.2–3.6)	0.157	1.5 (0.9–2.7)	0.578	0.7 (0.1–2.9)
	positive margin	0.153	0.7 (0.4–1.2)	0.814	1.1 (0.4–3.1)	0.359	0.8 (0.4–1.4)	0.738	0.8 (0.3–2.5)
	Pre-sRT PSA	0.103	1.6 (0.9–3.0)	0.404	1.6 (0.5–4.8)	0.226	1.5 (0.8–2.6)	0.335	1.9 (0.5–6.5)
	ADT	< 0.001	0.4 (0.2–0.7)	0.623	0.8 (0.3–2.0)	< 0.001	0.4 (0.2–0.7)	0.593	0.8 (0.3–2.1)
	LR	0.016	0.5 (0.3–0.9)	-	-	0.012	0.5 (0.3–0.9)	-	-
	NR	0.007	2.1 (1.2–3.5)	-	-	0.005	2.2 (1.3–3.9)	-	-
	RT to elective pelvics	0.107	1.7 (0.9–3.5)	0.945	1.0 (0.3–3.1)	0.125	1.7 (0.9–3.4)	0.916	1.1 (0.4–3.2)
	Dose to LR ≥ 72 Gy	0.426	0.8 (0.4–1.5)	0.072	0.2 (0.0–1.2)	0.361	0.7 (0.3–1.5)	0.086	0.2 (0.0–1.3)
	SUVmax in LR > median	0.071	2.0 (0.9–4.1)	0.050	2.6 (1.0–6.9)	0.087	2.0 (0.9–4.4)	0.150	2.1 (0.8–5.6)
	SUVmax in LR > 75% IQR	0.022	2.3 (1.1–4.6)	< 0.001	4.6 (1.9–11.5)	0.049	2.1 (1.0–4.4)	0.005	3.9 (1.5–10.1)
	SUVmax in NR > median	0.640	1.1 (0.6–2.0)	-	-	0.786	1.1 (0.6–2.0)	-	-
	SUVmax in NR > 75% IQR	0.895	1.0 (0.5–1.8)	-	-	0.799	0.9 (0.5–1.8)	-	-
Multivariate	SUVmax LR 75% IQR	0.022	2.3 (1.1–4.6)	-	-	0.049	2.1 (1.0–4.4)	-	-
	ADT	ns		-	-	ns		-	-
	LR	ns		-	-	ns		-	-
	NR	ns		-	-	ns		-	-

Abbreviations: *BRFS*, biochemical recurrence free survival; *ADT*, androgen deprivation therapy; *ISUP*, International Society of Urological Pathology Grading; *pT*, pathological T-stage; *pN*, pathological nodal stage; *sRT*, salvage radiotherapy; *PSA*, prostate-specific antigen; *LR*, presence of local recurrence; *NR*, presence of nodal recurrence; *Gy*, gray; *SUVmax*, maximal standardized uptake value; *HR*, hazard ratio; *95%CI*, 95% confidence interval; ns, non-significant bold indicates statistical significance

To analyze robustness and consider the confounding factor of short-term ADT, a sensitivity analysis with patients with a FU of > 12 months (*n* = 197, median FU 27 months IQR 20–43) was performed confirming the results showing no significant association of clinical and histopathological parameters with BRFS, presence of LR being significantly associated with favorable BRFS (*p* = 0.012, HR 0.5 95%CI 0.3–0.9), and presence of NR (*p* = 0.005, HR 2.2 95%CI 1.3–3.9) and SUVmax values ≥ 75% quartile in LR being associated with unfavorable BRFS (*p* = 0.041, HR 2.2 95%CI 1.0–4.6). Sensitivity analysis of patients with LR only and > 12 months FU (*n* = 83) showed a strong association of SUVmax values ≥ 75% in LR quartile with unfavorable BRFS (*p* = 0.005, HR 3.9 95%CI 1.5–10.1). See Table 2 and Fig. 3.

In multivariate analysis, SUVmax values in LR ≥ 75% quartile stayed significantly associated with unfavorable BRFS in the cohorts including all patients (*p* = 0.022) and in patients with > 12 months FU (*p* = 0.041). Presence of LR or NR in PET and administration of ADT was not significantly associated with BRFS in multivariate analysis. See Table 2.

### Time-dependent ROC

**Fig. 4:**
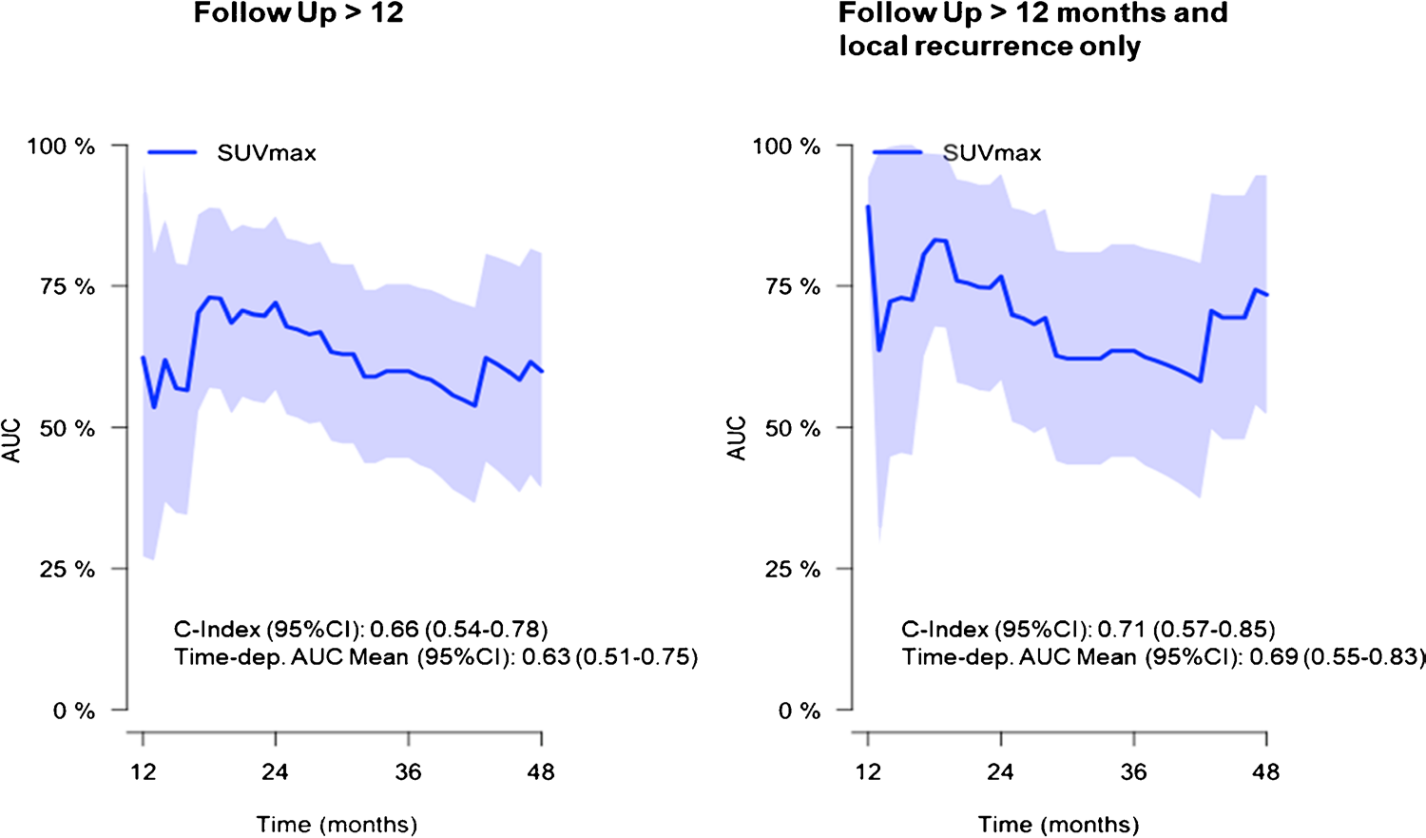
Time-dependent receiver-operator-characteristics (ROC) analysis of the maximal standardized uptake value (SUVmax). Concordance index (C-index) and area-under-the-curve values (AUC) with 95% confidence interval (95%CI) are shown. Transparent area demonstrates the 95%CI

Time-dependent ROC analysis of SUVmax values of patients with > 12 months FU and > 12 months FU and LR only yielded a concordance index (C-index) of 0.66 (95%CI 0.54–0.78) and 0.71 (95%CI 0.57–0.85), respectively. Prediction improved at 18 months FU. SUVmax cut-off values determined by the maximally selected rank statistic were 11.8 for all patients and 13.0 for patients with > 12 months FU in all patients and in patients with LR only, respectively. See Fig. 4 for details and supplementary Table 2 for further analyses.

## Discussion

SRT is the last curative option for patients with recurrent or persistent PCa after surgery, but heterogeneous responses demonstrate the need for improved and differentiated risk stratification and subsequently appropriate adaptions in disease management. While implementation of PSMA-PET led to relevant improvements in disease localization, with results of prospective trials pending, it is not yet clear whether the sole spatial information of tumor burden and the accompanying changes in treatment management have a relevant impact on progression rates. Considering that PSMA-PET-positive findings might only represent the “tip of the iceberg,” the additional biological information provided by this molecular imaging bears great potential. To our knowledge, this is the first large multicenter retrospective study to evaluate the potential of SUVmax values in PSMA-PET as a new biomarker in patients with PCa persistence/recurrence. The findings from this study suggest that SUVmax values may significantly contribute to identify patients who are at higher risk for progression after sRT and therefore might benefit from treatment intensification, guiding personalized treatment approaches.

In contrary to prospective trials investigating sRT, most of the patients included in our study are likely to be at advanced recurrent disease stages with 70% having a PSA ≥ 0.5 ng/ml prior to sRT and 59% having nodal recurrence. Therefore, the comparison with data of recently published randomized controlled trials [26–28] is limited. Nevertheless, sRT in fossa-confined patients yielded BRFS in 80% after 2 years. Considering slightly different definitions of endpoints, these results are comparable to freedom from biochemical failure in approximately 50–70% for patients with a pre-sRT PSA between 0.2 and 2.0 ng/ml in a large retrospective study of conventionally staged patients [5, 29]. BRFS rates for patients with node-positive PSMA-PET findings dropped dramatically to 65% after 2 years, which is in line with findings from a prospective trial, reporting 3-year BRFS rates of 45% with PSMA-PET-positive disease outside the prostatic fossa [30]. Our results thereby confirm that PSMA-PET findings are highly prognostic for BRFS.

In our retrospective analysis, presence of PSMA-PET-positive local and nodal recurrence positive on PSMA-PET were prognosticators for BRFS after sRT, whereas classical pathological and clinical parameters were not. Since Emmett et al. prospectively demonstrated the prognostic value of PET-positive findings, it is likely that they dominate established parameters in our cohort, since we included only patients with PSMA-PET-positive lesions. The improved BRFS associated with the presence of LR (HR 0.5, *p* = 0.016) is explainable by the dose escalation of PET-positive LRs yielding sufficient RT dose coverage, which was previously reported to be beneficial [10].

Despite favorable outcomes for patients with LR only, still nearly one-third of patients suffered from progression after sRT. In multivariate analysis, our results demonstrate a significant association of SUVmax ≥ 75% quartile (HR 2.3, *p* = 0.022) with unfavorable BRFS in this subgroup. Sensitivity analysis confirmed these results with a HR of 3.9 in patients with LR only and FU of > 12 months. These findings are in line with the biological understanding of PSMA, with high PSMA-expression being associated with more aggressive disease [31] and SUVmax correlating with PSMA-expression [32]. Therefore, patients with LR and high SUVmax values might represent a subgroup with more aggressive PCa, potentially suffering from micro metastases outside the prostatic bed at the time of imaging and therefore benefiting from intensified treatments. Cox-regression and ROC analysis furthermore suggest that SUVmax in LR might be a valuable prognosticator in patients with both LRs and NR. However, these results need to be interpreted carefully, since ADT is administered more often and for a longer period of time in patients with NR (44% in our cohort).

Whether extraction of additional radiomic features from PSMA-PET images enables identification of additional prognosticators [33] needs to be evaluated in future studies. However, implementation of SUVmax offers great potential in this scenario, since it is easily and non-invasively determinable with minimal resources and without additional costs and is not affected by interobserver variability [34]. AUC values of the time-dependent ROC analysis showed the best discrimination in patients with > 12 months FU and LR only. In an exploratory analysis, we calculated SUVmax cut-off value for optimal discrimination. In all patients, the optimized cut-off value was slightly lower than the 75% quartile (11.8 vs 12.8). In the subgroup with FU > 12 months, the cut-off value was, however, more similar (13.0). Thus, a SUVmax threshold of approximately 13.0 should be validated in future studies in PSMA-PET imaging. This being said, the role of SUVmax in these patient subgroups needs to be evaluated in future studies including new tracers to validate putative cut-off values and design studies, which evaluate treatment intensification such as extension of RT fields to elective nodes or intensified systemic treatments. To define optimal RT fields, patterns of metastases need to be vigorously analyzed. Intensification of systemic treatments in sRT is currently investigated by the FORMULA-059 RCT (NCT03141671). Keeping in mind that sRT should be initiated at low PSA levels, implementation of SUVmax into risk stratification might even be relevant in this scenario, with approximately 50% and 65% of patients having PET-positive findings at PSA values < 0.2 ng/ml and between 0.2 and 0.5 ng/ml [30]. Furthermore, administration of ADT was associated with favorable BRFS in the entire cohort in univariate analysis, but not in multivariate analysis. Additionally, ADT was not associated with BRFS in patients with LR only. Despite RCTs demonstrated a benefit of adding short-term ADT to sRT [35], our results show that these findings cannot directly be transferred into the PSMA-PET era. Our findings suggest that patients with recurrence confined to the prostatic fossa in PSMA-PET might not benefit from systemic, but rather from local treatment intensification. Considering recent results of the multicenter retrospective SPIDER 01 (Abstract OC-0607), we therefore evaluated the effect of doses ≥ 72 Gy to the PSMA-PET-defined local recurrence. We could not identify a statistical significant difference but a trend for favorable BRFS in patients with LR only, who received dose escalation ≥ 72 Gy (*p* = 0.086). However, this analysis is hampered by the relatively small number of patients receiving dose escalation and short follow-up. Future studies are needed to evaluate this aspect in depth.

Interestingly presence of NR was associated with significantly unfavorable BRFS (HR 2.1) but not SUVmax values in NR, suggesting that the additional biologic information provided by SUVmax values does not contribute to this patient subgroup, who already suffer from relevantly poorer prognosis. These patients might benefit from systemic treatment intensification, since despite dose escalation to PET-positive nodes, it is likely that sRT might not cover non-visible tumor spread.

Our study has some limitations. First, due to its retrospective design, protocols for PSMA-PET scans, sRT, and follow-up varied between centers and are prone to selection bias. Since the tracer kinetics depends on the time between injection and image acquisition, we want to point out that all scans were acquired in line with recent guidelines in all centers, but use of different PET scanners might affect comparability. Second, it is known that different reconstruction methods such as time-of-flight and point-spread-function have implication to SUV values. Most importantly, SUV values might be underestimated when taken from small lesions, i.e., sub-centimeter. In order to quantify this aspect, we analyzed SUVmax values with respect to lesion volume. We found a tendency of smaller SUVmax values for lesions < 1 ml, which could only be found in a minority of patients (*n* = 14). Thus, we are convinced that this limitation is very limited towards the overall results. Furthermore, there are many PSMA-ligands on the market and to date both ^68^ Ga-PSMA-11 and ^18^F-DCFPyL received FDA approval, with different pharmacokinetics and pharmacodynamics as well as isotopes, raising questions about the reproducibility of SUV values. In our study, we employed only ^68^ Ga-PSMA-11, in order to generate more standardized data [36]. Therefore, the different reconstruction algorithms between the different institutions pose the risk for deviation. In order to limit the inter-institution variability, we only employed PET/CT systems with EARL1 accreditations. However, this heterogeneity can also be regarded as a strength of our retrospective analysis as it makes the data more applicable in clinical routine when different scanners and reconstructions are used between different centers. Third, no central review of PET images was performed with potential differences in interpretation between centers. Fourth, the median FU is relatively short with a median FU of 24 months. Lastly pathological data from RPE was missing in up to 27% of patients, likely contributing to inferior Cox-regression results.

## Conclusion

Our study is the first to demonstrate that the SUVmax value is a promising new non-invasive biomarker to prognosticate outcome in patients undergoing sRT and recurrence confined to the prostatic fossa ± nodal recurrences in PSMA-PET. Its addition might contribute to improve risk stratification of patients with recurrent PCa and to guide personalized treatment decisions.

## Supplementary Information

Below is the link to the electronic supplementary material.

Supplementary file1 (DOCX 3578 KB)

## Data Availability

The datasets generated during and/or analyzed during the current study are available from the corresponding author on reasonable request.
