# Supplementary material for: The maximum standardized uptake value in patients with recurrent or persistent prostate cancer after radical prostatectomy and PSMA-PET-guided salvage radiotherapy—a multicenter retrospective analysis
Source: Eur J Nucl Med Mol Imaging. 2022 Aug 19;50(1):218–27. doi: 10.1007/s00259-022-05931-5 (PMC9668780; doi:10.1007/s00259-022-05931-5)
Supplement: Supplementary file 1 — Supplementary file1 (DOCX 3578 KB) [file 259_2022_5931_MOESM1_ESM.docx]

**Supplementary Material – Table 1. Salvage RT concepts**

| **Center** | **Dose to fossa (EQD2, α/β=1.6 Gy)** | **Indication (and field) for sRT to elective pelvic lymphatics** | **Dose to elective pelvic lymphatics and PET positive pelvic LN** | **Indication (and duration) of androgen deprivation therapy** |
| --- | --- | --- | --- | --- |
| Freiburg, Germany | Dose to fossa (R0): 64 Gy  Dose to fossa (R1): 66-68 Gy  Dose to local recurrence in PET: 68-72 Gy | pN+ status in surgery (whole-pelvis)  Positive pelvic LNs in PSMA PET/CT (whole-pelvis) | Elective pelvic lymphatics: 42.5-47.6 Gy  PET positive pelvic LN 50-60 Gy | pN+ status (0-24 months)  PSA prior to sRT >0.7 ng/ml (6 months)  Positive pelvic LNs in PET (6-24 months)  + individual decision (e.g depending on comorbidities) |
| LMU Munich, Germany | Dose to fossa: 66 Gy  (in case of whole pelvis RT: 64.75 Gy)  Dose to local recurrence: 70 Gy (in case of whole pelvis RT: 72.2 Gy) | pN+ status in surgery (whole- pelvis)  positive pelvic LNs in PSMA PET/CT (whole-pelvis) | Elective pelvic lymphatics: 46.75 Gy  PET-positive LNs: 64.75 Gy | Positive pelvic LNs in PET (6-24 months)  positive local recurrence in PET (6-24 months)  PSA prior to sRT > 0.7 ng/ml and Gleason 8-10 (6-24 months)  + individual decision (depending on comorbidities, Gleason, PSA prior to sRT) |
| TUM Munich, Germany | Dose to fossa: 68 Gy  Dose to local recurrence in PET: 82.88 Gy (SIB, 76.5 Gy in fractions of 2.25 Gy) | pN+ status in surgery, lymph node dissection with <10 lymph nodes, or risk for lymph node involvement of ≥20% (whole-pelvis)  Positive pelvic LNs in PSMA PET/CT (whole-pelvis) | Elective pelvic lymphatics: 47.04 Gy (50.4 Gy in fractions of 1.8 Gy)  PET positive pelvic LN: 60.76 Gy (SIB, 58.8 Gy in fractions of 2.1 G) or 65.71 Gy (SIB, 61.6 Gy in fractions of 2.2 Gy) | PSA prior to sRT >0.7 ng/ml (6-24 months)  + individual decision (tumor conference) |
| Bologna, Italy | Dose to fossa: 66-70 Gy | Positive pelvic LNs in PSMA PET/CT (half/ whole pelvis) | Elective pelvic lymphatics (+/-) including the PET positive nodes:  half-pelvis: 45-50 Gy  whole-pelvis: 45-60 Gy | Positive pelvic LNs in PET (6-24 months)  + individual decision (e.g depending on comorbidities |


**Supplementary Material – Figure 1: Interobserver variability** (A) shows a before-and-after plot of gross tumour volumes (GTV) delineated by reader 1 and reader 2. *=significant difference, p<0.001. (B) shows a before-and-after plot of SUVmax values extracted from the GTVs. ns=not significant

**Supplementary Material – Table 3: Time-dependent ROC analysis and maximally selected rank statistics for different cohorts.** Abbreviations: C-Index=concordance index, SD= standard deviation, AUC=area under the curve, SUVmax= maximum standardized uptake value, NR=presence of nodal recurrence

|  | C-Index  (SD) | Time-dep. AUC Mean (SD) |  |
| --- | --- | --- | --- |
| **All patients** |  |  |  |
| SUVmax 75%Quartile | 0.57 (0.05) | 0.58 (0.02) | 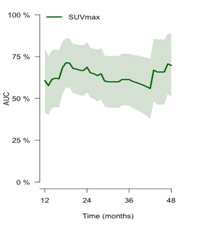 |
| NR | 0.55 (0.05) | 0.55 (0.02) | 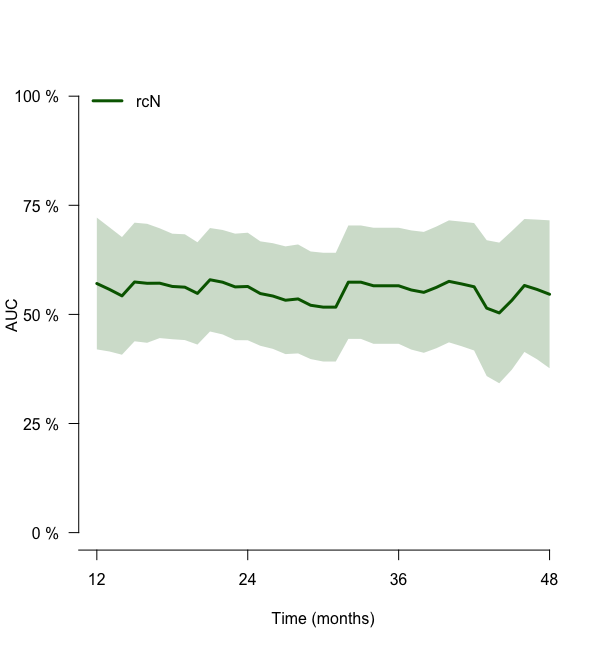 |

| **> 12 months Follow-Up** |  |  |  |
| --- | --- | --- | --- |
| NR | 0.56 (0.06) | 0.55 (0.03) | 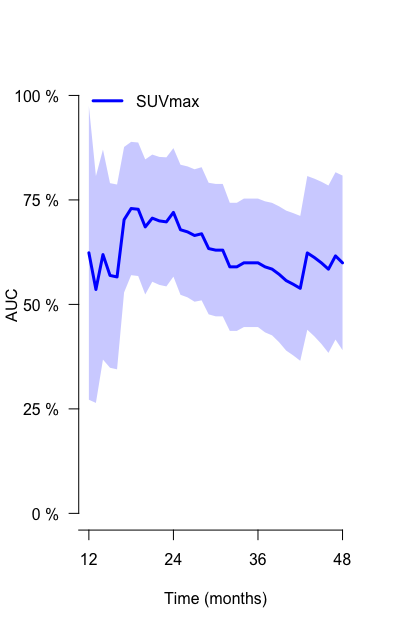 |

| **Patients with local recurrence only** |  |  |  |
| --- | --- | --- | --- |
| SUVmax 75% Quartile | 0.64 (0.06) | 0.64 (0.04) | 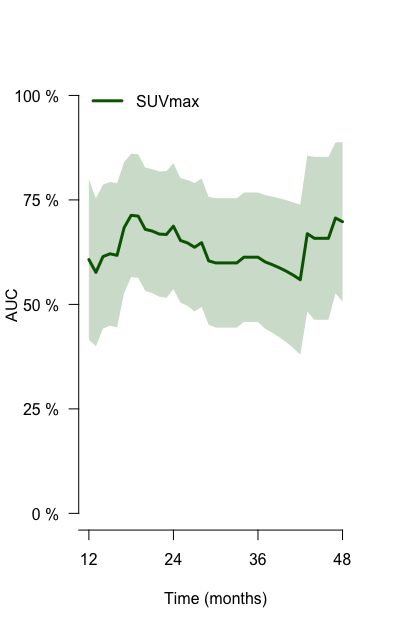 |
